# "If I have a cancer, it is not my fault I am a refugee”: A qualitative study with expert stakeholders on cancer care management for Syrian refugees in Jordan

**DOI:** 10.1371/journal.pone.0222496

**Published:** 2019-09-27

**Authors:** Manar Marzouk, Maureen Kelley, Ibtihal Fadhil, Slim Slama, Kajsa-Stina Longuere, Proochista Ariana, Gail Carson, Vicki Marsh

**Affiliations:** 1 Nuffield Department of Medicine, University of Oxford, Oxford, United Kingdom; 2 Ethox Centre and Wellcome Centre for Ethics & Humanities, Nuffield Department of Population Health, University of Oxford, Oxford, United Kingdom; 3 WHO Regional Office for the Eastern Mediterranean, Cairo, Egypt; 4 KEMRI Wellcome Trust Research Programme, Kilifi, Kenya; Makerere University School of Public Health, UGANDA

## Abstract

**Background:**

Noncommunicable diseases including cancer are widespread amongst the 5.6 million Syrian refugees currently hosted in the Middle East. Given its prevalence as the third leading cause of death in Syria, cancer is likely to be an important health burden among Syrian refugees. Against this background, our aim was to describe the clinical, ethical and policy decision-making experiences of health actors working within the current refugee cancer care system; the impact of refugee cancer care health policies on health care providers and policy makers in this context; and provide suggestions for the way delivery of care should be optimised in a sustained emergency situation.

**Methods:**

From April-July 2016, we conducted in-depth interviews with 12 purposively sampled health officials and health care workers from the Jordanian Ministry of Health, multilateral donors and international non-governmental organisations. Data were analysed using a framework analysis approach to identify systemic, practical and ethical challenges to optimising care for refugees, through author agreement on issues emerging from the data and those linked more directly to areas of questioning.

**Results:**

As has been previously reported, central challenges for policy makers and health providers were the lack of quality cancer prevalence data to inform programming and care delivery for this refugee population, and insufficient health resource allocation to support services. In addition, limited access to international funding for the host country, the absence of long-term funding schemes, and barriers to coordination between institutions and frontline clinicians were seen as key barriers. In this context where economic priorities inevitably drive decision-making on public health policy and individual care provision, frontline healthcare workers and policy makers experienced significant moral distress where duties of care and humanitarian values were often impossible to uphold.

**Conclusions:**

Our findings confirm and expand understanding of the challenges involved in resource allocation decisions for cancer care in refugee populations, and highlight these for the particular situation of long term Syrian refugees in Jordan. The insights offered by frontline clinicians and policy makers in this context reveal the unintended personal and moral impact of resource allocation decisions. With many countries facing similar challenges in the provision of cancer care for refugees, the lessons learned from Jordan suggest key areas for policy revision and international investment in developing cancer care policies for refugees internationally.

## Introduction

Seven years of conflict in Syria has resulted in the displacement of over five million Syrians. The majority are hosted by neighboring countries; Lebanon, Jordan and Turkey [1]. Currently, there are approximately 660,000 Syrian refugees registered in Jordan, which represent a significant percentage of Jordan population of 9,456,000 [2,3]. Unlike other humanitarian crises, the reported data from refugee settings in Jordan has shown that Syrian refugees are not dying from infectious diseases, but rather a lack of access to care for noncommunicable diseases (NCDs) [4]. Before the war, cancer was the second leading cause of death in Syria and it remained high during the war; as of 2012, cancer was the third leading cause of death after heart disease and injuries [5]. While robust evidence on cancer rates in Syrian refugees is not available, initial estimates point to significant burden of disease in this population [6,7]. Moreover, the prolonged nature of the Syrian refugee crisis has prompted the need for a change in humanitarian policies to meet refugees’ longer-term healthcare needs, to which NCDs, including cancer, are an important contributor [8,9]. Our qualitative study with experts working in refugee cancer clinical care and policy aimed to understand practical and ethical challenges experienced by this group, and identify areas in which policy and services could be strengthened.

### Cancer care in Jordan for nationals and refugees

Jordan is currently classified as an upper middle-income country by the World Bank, and as part of a low-and-middle country (LMIC) region by World Health Organisation [10,11]. In Jordan, almost all nationals with cancer have full private health insurance coverage. Those who are unable to afford health insurance can apply for a ‘vulnerability exemption’ from the Jordanian Royal Court, known as “Diwan Malaki” [12,13,14]. Access to cancer care for Syrian refugees in Jordan does not follow the same model. While cancer care for Syrian refugees was initially subsidised to the same level as for insured nationals, this was changed in 2014 to allow subsidies only to the level of uninsured Jordanians, and only for those refugees registered as official asylum seekers (possessing an asylum seeker certificate from the United Nations High Commissioner for Refugees (UNHCR) and a Ministry of the Interior Service Card from the Ministry of Health). While these subsidies are greater than would be available to non-refugee foreigners in Jordan, the costs are generally prohibitive for Syrian refugees. According to the UNHCR’s Vulnerability Assessment Framework Baseline survey, approximately 86% of Syrian refugees living in the Jordanian community live below the poverty line and have restricted access to available services [13,15]. It is estimated that at least 160,000 Syrians have left camps without the authorisations needed to register as asylum seekers, leaving them unable to register for healthcare subsidies. For some, this is reportedly due to a reluctance to register with UNHCR or local police [16]. Similarly, access to work is restricted by a need for work permits, which are difficult to obtain due both to their cost (approximately USD 400) and the need to pass a background security check by the Ministry of the Interior [17].

### Addressing the treatment gap for cancer among refugees

One potential factor underlying Syrian refugees’ difficulties in obtaining access to health care is that Jordan did not sign the 1951 Geneva Convention, which would oblige it to provide refugees with the same treatment as nationals [18]. Absent this national commitment, it remains unclear who should take responsibility for addressing the gap in cancer care for refugees (Article 23, 1951 Convention and its protocols, 1967) [18]. However, recent developments in the health resource allocation policies of several key healthcare actors in Jordan have marked an improvement in refugees’ access to cancer care services, driven at least partly by recognition of the high prevalence of non-communicable diseases (NCDs) among Syrian refugees, including cancer, cardiovascular problems and diabetes [6]. Given this situation, several humanitarian organisations have expanded their activities to include NCDs. For instance, in 2014, Médicins Sans Frontières (MSF) added five specific NCDs to their care policies: asthma, cardiovascular disease, chronic obstructive airways disease (COPD), and type 1 and 2 diabetes [19].

In a similar way, the UNHCR has established an ‘exceptional care committee’ (ECC) as a triage system to identify eligible Syrian refugees for whom funding and referral for treatment at cancer care centres in Jordan could be supported under UNHCR funding [20]. The ECC consists of three members—a UNHCR health officer, a local oncologist and a medical consultant—who meet monthly to review medical reports of potential referral cases and make decisions based on disease type and stage, prognosis, treatment costs and funding availability. The committee works through the Jordan Health Aid Society (JHAS), an international non-governmental organisation (NGO) that runs six clinics in Jordan in areas with a high concentration of Syrian refugees (in Amman, Irbid, Mafraq, Zarqa and Al Ramtha, and at Zaatari camp), and a further mobile medical unit to cover south Jordan [21]. JHAS can refer cancer patients from refugee camps or the wider community to the ECC for decisions about access to UNHCR funding, which will be based on criteria including cost and the availability of appropriate specialised care at each institution. Where considered eligible, patients are referred by JHAS to one of the three main Jordanian cancer treatment institutions.

Whilst recent efforts to address the burden of NCDs in refugee populations have been encouraging, the current humanitarian response strategies for NCDs in Syrian refugees remain largely based on experiences from refugee camps in sub-Saharan Africa where communicable diseases are significantly more common [6]. There remains a need to strengthen cancer care services for refugees from countries in the Middle East, where health needs are notably different [6]. This is particularly important in the Syrian case given the prolonged displacement periods experienced by refugees and the estimated high prevalence of cancer in this population [6,7].

However, the literature on cancer care in refugee settings is limited. Illustratively, in developing this study, we conducted a scoping review of the literature through PubMed and websites of the World Health Organization, Jordanian Ministry of Health and UNHCR. Using search terms (Refugees* OR Asylum Seeker* OR Displaced) AND (Cancer OR Leukemia OR Leukaemia), 32 papers were found on PubMed, over 75% of which concerned services for refugees in the USA. Since 86% of refugees are hosted in low-to-middle income countries (LMIC) [22], the current focus in the literature (both published and grey) on cultural barriers for refugees in accessing cancer care in high-income countries is not widely informative [23].

To contribute to the evidence base to inform cancer care systems for refugees in the Syrian-Jordanian context, this paper reports on an in-depth qualitative case study conducted with key expert stakeholders in cancer care for refugees in Jordan. Against this background, our aim was to describe the clinical, ethical and policy decision-making experiences of health actors working within the current refugee cancer care system; the impact of refugee cancer care health policies on health care providers and policy makers in this context; and provide suggestions for the way delivery of care should be optimised in a sustained emergency situation. Specifically, we explored the ways in which practical and ethical challenges arise, the rationale and strategies behind responses to those challenges, and the ethical implications of refugee cancer care policies in this resource-limited context. Participants offered valuable and timely insights into the current situation, including challenges, and ideas for improving cancer care systems for Syrian refugee populations hosted within Jordan, suggestions which may inform future policy development.

## Methods

Given the need for deeper insights into the specific context and unique challenges for cancer care management within the Syrian-Jordanian refugee crisis, we designed a qualitative expert stakeholder study to describe the views and experiences of a group of experts in Jordan on cancer care policies and practice. Participants included policy makers, managers and providers working in the Jordanian health care system and with humanitarian aid organisations working with Syrian refugees. Data were collected from 12 such stakeholders between April and July 2016, purposively sampled from different types of actors working in the field of cancer care for refugees, including: i) The Jordanian Ministry of Health; ii) UNHCR, as a multilateral donor; and iii) two differing international non-governmental organisations, MSF and JHAS; where MSF has an international focus and JHAS activities are based entirely in Jordan (S1 Table).

Participants were identified through the literature review described earlier, the lead author’s prior knowledge of health actors in the Syrian refugee setting, advice from the WHO EMRO and snowball sampling based on recommendations from prior interview participants [24–25]. The research team decided to focus on a participant group with direct experience of policy and care provison group in the Syrian-Jordanian refugee setting, given the aims of the study, and fifteen invitations were sent by email to relevant potential participants. While 13 agreed to participate, one policy maker was subsequently not contactable. The invitation to participate included information on the study purpose and process, including the right to withdraw at any time, and in the national language [26]. We assessed the sample size as appropriate to the narrow scope of inquiry around a very particular problem with few ‘expert voices’.

The lead author conducted all in-depth interviews using Skype, each lasting between 30 minutes and one hour (see S3 File. Question Guide, English & Arabic, Cancer Care). Since the lead author is a native Arabic speaker, all participants were given the choice of being interviewed in Arabic or in English, and one chose the former. Interviews were audio recorded, and translated and transcribed in English by the lead author.

Data were analysed using a framework analysis method, drawing on deductive (*a priori*, or from the research questions) and inductive (emerging from the data) approaches [27]. MM, VM and MK read all the transcripts and independently developed draft coding frameworks before discussing and agreeing on a version used by MM for coding the dataset. Transcribed interviews were uploaded to NVIVO 11 software to support the coding process. Over the course of data analysis, MM, VM and MK held several meetings to discuss ‘fit’ between the data and the coding framework and adapt the latter where needed, including through the development of new codes to account for emerging themes. This group recognised and drew on their sometimes overlapping roles as a Syrian public health professional, USA bioethicist based in the UK and UK public health researcher with a strong focus on global health ethics. A particularly important emerging theme for this paper, for example, came from data on the moral distress of policy makers and health providers in balancing the challenges inherent in their work, as described in the findings section. During this process, these authors sought to explore possible issues around the translation from Arabic to English as part of the data analysis and interpretation, in terms of language and cultural influences. Through this discursive and iterative process, we assessed that a reasonable level of saturation of the themes of interest had been reached [28–29].

### Ethical considerations

Ethical approval for this study was obtained through the University of Oxford Tropical Research Ethics Committee (OxTREC). All participants were sent information sheets and consent forms on the study ahead of the interview, and gave signed consent for their involvement. The information sheet is included in S4 File. Participant Information Sheet. Given the small number of expert participants from such a specific context, and potential sensitivities in the topics discussed, all data were anonymized by removing direct links to employing organisations, as agreed in the informed consent process. In addition, to support frankness in sharing information during these interviews, and in keeping with the ethics approval given by OxTREC, we reassured participants that data collected during the study would not be made available more widely outside this study. Subsequently, while quotes from one participant were removed, all the illustrative quotes in this manscript were approved by participants for inclusion.

## Results

In this section we present our findings across three *a priori* or emerging and interrelated themes: participants’ perceptions of key challenges for the development or enactment of effective policies on cancer management for Syrian refugees; the personal strategies that individuals have developed to navigate these challenges; and the ethical dilemmas arising for those involved in cancer care in this context.

### Challenges for cancer policy development & implementation

#### Perceptions of a decreased international commitment

At a global level, the international community was described as having an important role in helping countries manage the economic burdens of the Syrian refugee crisis on neighbouring countries [30]. While this complex situation is well recognized internationally, participants expressed a feeling of being ‘overlooked’ by the international community. Recognition of the limitations of the Jordanian health system and the rapid rise in demand for health services from refugees due to the Syrian crisis were cited as reasons for the 2014 Jordanian government policy changes on access to health care for refugees [31]:

“*In the beginning*, *it [subsidized cancer care] was supported internationally* *…* *In recent times the situation is getting difficult…and because the international community are not fulfilling their promise of support for Jordan, that is the main cause I think for reducing the services…Also, because it [influx of refugees] has happened in a very short time, increasing the Jordanian population by about 15% to 20% of the population in 2–6 years. The infrastructure is not ready to absorb this large number, this is not only for the clinical or health sector.”*

(*D1, PM*)

Given this funding shortage, policy makers described using cost-saving referral policies, attempting to refer adult patients to the least expensive public sector hospitals first, followed by private sector hospitals, a system that generally mirrored levels of specialist care available. By contrast, children were considered a higher priority, and were usually referred directly to the most expensive specialist private facilities at the King Hussein hospital.

#### Lack of available data for planning

At a national level, one of the main challenges raised for developing cancer care policy in Jordan was the recognised scarcity of data about cancer prevalence amongst Syrian refugees and available health services in the country. This was seen as hindering evidence-based strategic planning and advocacy for further funding for these patients:

“*Data collection…would help us to understand challenges faced by the community and prevalence as an organization. We can institute some programmatic changes within our programme to address the needs based on actual figures, and scientific basis… And push other health actors toward what it is mostly needed on the ground*. ”

(*A2, PM*)

“*If there is a documented official number of cancer cases, you can demonstrate this to the international community. It would be helpful to advocate…and get better funds*”

(*D1, PM*)

#### Financial costs and linked bureacratic challenges for refugees

According to UNHCR’s vulnerability assessment Framework Baseline survey, around 86% of Syrian refugees in the community live below the poverty line and have restricted access to available services [15]. As such, Syrian refugees with cancer face substantial financial barriers for accessing treatment. As mentioned, policies for access to cancer care for Syrian refugees, either in refugee camps or in the wider community, differ from that available to Jordanian nationals. As described by policy makers in this study, subsidies initially provided for Syrian refugees at the same level as for all nationals were adjusted in 2014 to match the level of uninsured Jordanians, and only for those refugees registered as official asylum seekers. While these subsidies are greater than would be available to non-refugee foreigners in Jordan (refugee subsidies reduce costs to about 30–60% of those for foreigners), the costs are generally prohibitive for many Syrian refugees.

Experts reported additional bureaucratic challenges facing refugees seeking official asylum seeker status, including the need to obtain a UNHCR asylum seeker certificate and a Ministry of the Interior Service (MOI) Card from the Jordanian Ministry of Health in order to register for health care subsidies [13–15]. It is estimated that since September 2016 at least 160,000 Syrians left Zaatari camp without the authorisations needed to register as asylum seekers, including through a reluctance to register with the UNHCR or local police. As reported, reluctance stemmed from an inability to afford the costs of registration, not having the required documentation and, in some cases, a fear of approaching authorities or the police [15–17].

Further, financial challenges are worsened by the fact that many refugees would have difficulties in finding work to earn an income since mandatory work permits were described as difficult to obtain, including for reasons of cost (around USD 239–521) and the need to pass a background security check by the Ministry of the Interior [16–17]. Since January 2015, refugees who left the camp without appropriate authorisations have been ineligible for MOI cards and must pay for health care at a foreigner’s rate [16–17].

Together, these conditions for accessing health care subsidies and gaining employment can introduce often insurmountable obtacles to patients’ access to cancer care. As a health provider noted:

“*For us [Jordanians]*, *what it is paid is very trivial*. *But for them*, *as refugees*, *they see it as a big amount*. *Because they don’t have income*, *they don’t have anything*..*”*

(*A4,HCP*)

Of note, some health providers within international non-governmental (INGO) centres described being able to act with more flexibility in practice, responding to the medical needs of individual patients:

“*We don’t care whatever documents they have. If they have any or not… We rely on what they say. You can just register and get your appointment. We don’t care if he went to the camp, or was smuggled out the border, what we care about is he is in need and we need to help him*.”

(*A1, HCP*)

At the same time, as described further below in the section on ethical dilemmas, trying to work around bureaucratic policies sometimes generated considerable tension for health providers.

#### Physical access to care and referral policies

Physical access to health centres presented a futher challenge to some refugees’ ability to access cancer treatment. Refugees living inside camps were particularly affected since they would only be given permission to leave the camps for few hours, and applications for authorization had to made ahead of time. Since referral centres were often situated at a distance from refugee camps, travel time, queueing, having tests, and waiting for treatment could often exceed the time allowed. Yet breaching these restrictions could lead to deportation:

“*Sometimes they don’t get back to the camp exactly in the same time, so if any of the police find any of these refugees outside the camp or his permission ended, they send him back to the camp or to Syria, if they are not convinced of the story why he is away from the camp*.”

(*A1, HCP*)

Health staff at referral centres recognized this predicament, and described prioritizing patients from camps to help them to return on time:

“*Sometimes we give an exception for those patients living inside camps. We try to finish them earlier because you know the transportation between cities is so hard*.”

(*A3, HCP*)

Participants also noted that routine health services–as opposed to cancer specific care–are generally more accessible for those living in camps:

“*I think in the camps it is a lot easier for people to access services because in the camps all the services are available for all those in the camps*.”

(*A5,PM*)

At the same time, some policy makers described ways in which the refugees living inside camps were seen as more vulnerable in relation to health needs than those outside. They observed that this was in part due to an additional bureaucratic hurdle for community-based refugees in accessing health care; this group would need to meet an additional “vulnerability” criterion, assessed by UNHCR staff using a ‘Vulnerability Assessment Form’, a survey tool to assess the vulnerability of urban refugees and decide whether they are eligible for financial support.

### Personal strategies for cancer care seeking, referral and support

Given the financial and logistical barriers described above, participants reported significant difficulties in trying to support Syrian refugees with cancer in accessing health care. Strategies for accessing and paying for care often fell to patients and their families, and frontline health providers.

#### Patient and family strategies

At the beginning of the crisis, some refugees were able to afford some forms of (subsidised) cancer treatment, but in time, their money ran out. For refugees who were ineligible for subsidised care, the social structure within the Syrian community played an important role in meeting costs, particularly for those who had relatives in the Gulf and high-income countries.

“*Some patients … have siblings or relatives working for the UAE (United Arab Emirates), US, UK so they can send them part of the treatment [cost]*.”

(*A1, HCP*)

This financial support sometimes comes from pooling funds from a range of small donations, in response to requests for help:

“*They collect money from relatives, friends, network and they try to pay for hospital to cover the cost … The whole process is about gathering money, and collecting money from people in the Gulf and immigrants outside*.”

(*A3, HCP*)

At the same time, asking for money was not seen as an acceptable practice by most Syrians, but rather as humiliating and undermining their dignity.

“*Poor Syrian patients, if they are diagnosed with cancer, they need to beg for money and collect donations. The problem is some people don’t accept the idea of begging for money, so they may leave they family members without treatment until they die or at hospital asking them to take fluid until they die*.”

(*A3, HCP*)

For this reason, some refugees would choose to buy medication from a pharmacy without medical consultation, or fail to continue treatment, often leading to an earlier and more distressing death than might otherwise have been the case.

“*I have seen many patients; they are going directly to the pharmacy to get their medication*. *Because they can’t afford even to have consultation*.”

(*A2, PM*)

#### Health provider strategies, advocacy, and distress

All health providers interviewed expressed feelings of helplessness and powerless in the face of the challenges described. These emotions often led to personal intiatives to try to fill the policy gap for Syrian cancer patients. Examples included nurses and doctors informally following up with patients who could not access health centres at home, and “making exceptions”, for example, by conducting lab tests, while actively seeking other solutions:

“*There is one patient…he is not able to leave his home*, *he has really limited money*, *and it is really expensive tests to do*, *so there was continuous conversations with the staff around him…to try to figure [out] other options… we agreed that we do the test because he hadn’t done it for a quite a while… until we find another option for him*.*”*

(*A5, PM*)

Health providers described an informal referral pathway that, over time, served as a means of advocating for regular diagnostic referrals, and the extension of services by organisations such as MSF to include cancer within their NCD programme.

“*Sometimes*, *I even become an annoying source for my managers*. *Keep calling them and telling them that those patients need lab tests for cancer session*.*”*

(*A1, HCP*)

Health providers’ personal concern for their cancer patients also led to the establishment of an informal donation platform to support individual cases. This activity was often not supported by managers, given concerns that patients and their families would misunderstand the source of this support as being institutional.

“*Sometimes*, *we could as a team gather funds to treat a patient for a month*. *But our organisation says we don’t want such thing inside the organisation structure*, *because they are worried that the patient thinks that this fund is from the organisation itself*.*”*

(*A4, HCP*)

Another intiative involved linking patients with other NGOs and donors providing cancer care, and using personal connections to facilitate access to governmental health care.

“*Mostly I try to contact other NGOs if they can provide any cash assistance*. *Because this is the only way they can manage to afford a part of the cost of the treatment… I had some connections with [someone in] the government who can give them 100% discount*. *Sometimes when I see that the case is really complicated and no one is helping them*, *I have to call these connections to provide help*.*”*

*(A1*, *HCP*)

When these initiatives failed, health providers nonetheless continued to provide emotional comfort and social support for patients, often outside of normal working hours, to ease patients’ suffering.

“*We do not have their medication or the diagnostic procedures and we can’t cover their admission to hospital or rehabilitation or palliative care centers in Jordan*. *But we try just to support them psychologically or socially*.*”*

(*Interview A3, HCP*)

#### Policy makers’ strategies and advocacy

Policy makers also strongly recognized the importance of the unmet needs for Syrian refugees around cancer care, although they often viewed this challenge in a wider context.

“*We are in a middle income country… so [cancer] is a common chronic disease*. *And most [refugees] are coming with western diseases*, *and many they had treatment beforehand and came here and completely stopped the treatment*. *Which puts them backward in management of their cancer* …*”*

(*A5,PM*)

Some policy makers described efforts to accelerate the resettlement of certain categories of vulnerable refugees with cancer to a high income country, particularly to Scandanavian countries and the USA. This pathway is used by UNHCR policy makers mainly for cases with good prognosis, where lack of funding is the main barrier to subsidising treatment. This route depends on the willingness of host countries to resettle refugees, an uncertainty given that the rate of refugee acceptance internationally has decreased in recent years [22,32].

“*If the prognosis is good but it is going to be high cost*, *this type of case we will submit urgently for resettlement to a third country*. *For instance*, *we had a child with acute lymphocytic leukemia*. *…[W]e submitted urgently to resettlement countries*, *one of the Scandinavian countries*, *and he departed in within a month*.*”*

*(B2*, *PM)*

### Moral distress in refugee cancer care

The challenges experienced by health providers and policy makers in supporting Syrian refugees with cancer, and attempts to personally address the gaps in care, generated a shared sense of personal and moral distress. Moral distress is a term used to capture feelings of frustration, worry, or sadness resulting from knowing what is the morally right or best course of action but being constrained by circumstances or resources in being able to carry out one’s felt moral obligations [33]. Health providers reported frustration, emotional distress and burnout at not being able to carry out one’s professional and personal duty of care given constrained resources. Policy makers reported distress in having to make allocation decisions on the population level that they knew to be unfair for particular patients.

#### A frustrated sense of a duty of care

Not surprisingly, health providers expressed strong frustrations and sadness around their inability to offer cancer care to refugees. The source of frustration, as described, included a strong sense of professional responsibility to provide care, and a sense of duty rooted in a common humanity. The latter was often described as being underpinned by faith and cultural and historical identification with their Arab neighbours.

“*I know one patient died because he didn’t have the money to pay for the hospital and he died…*.*It is very bad feeling*, *he is a human*, *he is a Syrian*, *also for me as a Jordanian man*, *Arab man*, *he is a brother*, *I couldn’t support him*, *I couldn’t find a solution for him*. *Sometime*, *it is something related to my belief*, *my religion*, *my attitude*. *It is very bad for me*.”

(*A3,HCP*)

“*Eventually we are human if I can say that*. *And I don’t know*, *I feel that everyone has his role in this world*, *at least to help anyone*.

(*A1, HCP*)

Participants also expressed a wider sense of unfairness when reflecting on refugees’ lack of access to cancer care and inconsistencies and lack of coordination in resource allocation decisions across humanitarian organisations.

“*If I have diabetes I can access services; if I have cancer I can’t access any services*. *This is type of discrimination*. *If I have a cancer*, *it is not my fault I am refugees*, *and if I have diabetes it is not my fault*, *so services should be available for everything*.*”*

(*A2, PM*)

“*When it is winter*, *all NGOs distribute blankets*, *so when you go to a house of refugee*, *he has 5000 blankets and nothing to eat*. *Some NGOs can provide health care… another cash*, *some take care of cancer*, *just they have to cooperate with each other and to be more active*.”

(*A1, HCP*)

The sense of unfairness was heightened when health providers considered how commonplace the need had become. As one provider noted, *“Almost each house has cancer patients” (A4)*.

#### The ethical and political challenges of resource allocation

While health providers spoke most emotionally about a sense of failing in their duty of care for cancer patients, policy makers recognized the wider policy and political implications of expanding care and discussed the challenges of navigating policy limitations in emergency contexts, conflicting organizational policies, donor influences and national and international politics. When setting health policies, policy makers attempted to take into account the customary standard of care that refugees had accessed before fleeing their country. However, priority was often given to ensuring the availability of health care services to Jordanian nationals in Jordan and this placed a substantial financial limit on levels of care provided for refugees. Several policy makers noted the historical influence and lessons learned by international agencies from refugee situations in Africa.

“*As an emergency organization*, *if you look at Africa*, *South Sudan*, *every month there is an emergency*, *people dying from simple treatable conditions like malaria*, *measles*, *malnutrition*. *So such things*, *from a public health perspective*, *you need to prioritise*, *where you can maximize the impact by allocating your resources*, *versus investment [in]… diseases which require long costly sophisticated complicated treatment*….”

(*A2, PM*)

Experts reported that the main focus for donors is the cost effectiveness of NCD care, viewing the number of patients treated per monetary unit as a primary measure. The central ethical dilemma faced by policy makers was that between meeting the overall public health interests versus the interests of individual refugees.

“*If you have 50*,*000 dinars*, *would you spend this on vaccinating 10*,*000 children or will you spend this on one case of cancer? Clearly… you will spend it on vaccinating 10*,*000 children because this is going to save more lives*, *prevent more infections*.”

(*B2,PM*)

Needing to prioritise within a limited budget, policy makers felt forced to make difficult decisions about access to care and appreciated what was at stake in this trade-off.

*Once we have the choice to intervene for a life saving case*, *if you have a case with good prognosis what would you choose? … You have to be tough in certain points … It is definitely depends on the availability of budget and donor countries*, *and this is really challenging*.”

(*B1,PM*)

Particular consequences of limited funding in short term cycles were noted for conditions requiring long term planning, such as cancer:

“*I think because a lot of funding is changing… it is short term*, *then there is no funding for that [cancer]*. *So*, *the [health care] organizations are providing care*, *then their funding runs out*, *then you need to find*, *where is the next one?*”

(*interview A5*)

These decisions were being made against the backdrop of an evolving crises in Syria: the essential needs of refugees were increasing daily, while international funding support was decreasing.

“*The other issue is how much the donor is interested to support this high cost treatment*. *To consume a lot amount of money to support a few hundreds cases*, *while there are another few hundreds or thousands waiting for more essential services…*”

(*B3,PM*)

Lack of funding forced some policy makers to make sometimes tragic decisions, such as witholding on-going chemotherapy for patients in the knowledge that their health would deteriorate

“*Yes we may stop*, *mmmm not stop let’s say it is harsh word … We put some cases on hold until we get funds… This is another disaster*, *because cases will deteriorate…”*

(*B1,PM*)

One policy maker noted the importance of respecting patients’ dignity as a key consideration in resource allocation policy. For example, since MSF were offering uncomplicated surgical procedures in hospitals in northern Jordan, a referral for breast cancer to that facility for excision was seen as reasonably cost-effective.

“*It is complex but …if I am a female refugee and have a small breast lump that is detected as a … cancer*, *if it is a small surgery… that can help prolonging my life… that can restore my dignity*, *you know this is [the] very overall objective of all humanitarian organizations*, *so why not*?*”*

(*A2, PM*)

#### Impact on emotional wellbeing of providers and policy makers

Nearly all health providers and policy makers in our study described feelings of helplessness and frustration in the face of what they perceived to be difficult, unfair, and even tragic decisions.

“*Sometimes*, *I feel like I want to cry…it is huge conflict in your feelings*. *But mostly you are annoyed*, *because you can’t do whatever you want and you can’t give the help you see… Because the general view of NGOs [is that] cancer patients will cost 10*,*000 for each session [whereas] we can cure for example 50 [other] patients…They have to pay attention to the human…they are helpless*, *they can’t go out of the camps without permission*, *they can’t work*, *and getting a permission needs a week and they are spending lots of money on things that they don’t even need*.*”*

(*A1, HCP*)

Some participants described the importance of even small gestures to assist or advocate for patients; doing something was better than nothing:

“*Outside the clinic*, *we sometimes try to collect money for them …I need to do that to feel better when I face a Syrian cancer patient who asks me for help*”

(*A3, HCP*)

The most difficult decisions involved withholding the treatment for patients, generating the very difficult task for health providers of communicating this information to sometimes desperate patients and families:

“*Ahhh what do I feel (20 seconds silence)*. *For sure the patient will feel disappointment…they feel depressed*. *So what do you think they feel when you inform them that there is no institution covering the cancer?*”

(*A4,HCP*)

“*It is frustrating because you want to be able to help them*, *but…it is not within our capacity to be able to provide that* .... *It is hard to tell patients*, *we can treat your diabetes but we can’t treat your cancer....*.”

(*A5,PM*)

## Discussion

Cancer continues to be a neglected NCD in refugee crises. Competing priorities and lack of resources in LMIC, where most of refugees settle, continue to priortise infectious diseases. This is especially true in contexts where the cost of cancer care is high and not subsidized by the national health system. Jordan offers a rare example of an attempt by humanitarian organisations and a host country to provide cancer care. To the best of our knowledge, this study is the first to report the views of frontline health care providers and policy makers from cancer care for refugees in a LMIC and offers valuable lessons for considering the often unintended consequences of refugee and humanitarian health resource allocation policies, and inform thinking about ways in which these could be strengthened.

We have identified a number of challenges for developing cancer care policies for Syrian refugees in Jordan. The first is the limited data on the prevalence of cancer among the refugee population, which is needed for long term planning and strategy development for cancer care. A second factor has been a shift in financial support from the international community for Jordan. A third factor is the high cost for cancer treatment in comparison to other health programmes such as vaccinations, for which it is possible to treat more patients per monetary unit; this can make cancer less attractive to donors, leading to reduced funding [34]. Fourth, even when services are available, patients’ access is often hindered by official rules and restrictions related to refugee status, including restrictions on movements outside the camps and required documentation from security and police, such as a MOI card and an asylum certificate, for accessing available services [35].

For refugees who do not meet the eligibility criteria for subsidised care, family networks and contacts with wealthier relatives played an important role in filling the cancer care gap by covering the treatment cost- fully or partially. While those with less wealthy contacts or who felt humiliation in begging for treatment are often left without alternatives, leading to often tragic consequences of skipping treatment, resulting in early death.

Not surprisingly, such difficult cases have been emotionally frustrating and distressing for both frontline health providers, whose duty of care clashed with lack of funding and current cancer policies for refugees, and policy makers whose decisions had a direct impact on these patients and their families. This finding echoes recent work done with health professionals working in an austerity context, where similar distress was reported in the inability to care for patients in an appropriate and dignified way as required by a deeply felt sense of professional duty [36]. Participants in this study also expressed a sense of failing to meet the obligations within a shared humanity, or cultural brethren. One of the positive unintended consequences of policy gaps in refugee cancer care, were the many accounts of personal advocacy on the behalf of patients to gain access to care. Such efforts went beyond individual initiatives, as illustrated by the donation network developed within MSF to cover costs for some patients, resulting in a referral pathway for diagnostic tests and home visits for cancer patients, and the referral for emergency resettlement for high cost cases with a good prognosis. It is an example of the way in which strongly felt ethical obligation sparked creative solutions. An important next step is to assess feasibility and sustainability of these pathways at scale.

Distress and motivations toward advocacy were not only experienced by frontline health providers. Policy makers also struggled to balance broader public health needs and the needs of refugees with cancer with limited resources, and attempted to find ways to improve access. Policy makers justified tough decisions by the need to benefit the majority whose health needs may be less costly. Nevertheless, these justifications did little to lessen the emotional burden on health staff on the frontlines of patient care. Again, this experience is echoed in similar contexts. A study on Greek health workers linked shortages of medical supplies with emotional exhaustion and depersonalization [37]. A central strategy to cope with frustration and helplessness was to help these patients in whatever way possible. Some of the stories shared suggested a need for emotional and mental health support for health staff working with refugees.

Participants described a particularly distressing vicious cycle around delayed diagnosis. The absence of early cancer detection for refugees was seen by clinicians as a failure to meet the basic duty of care for cancer patients. Lack of screening and timely diagnosis also meant that cancers worsened before finally being diagnosed, making those patients more costly to treat with limited resources for refugees. In fact, late stage cancer cases are automatically ineligible for subsidised cancer care by the ECC committee. Patients with treatable cancers are often being referred for palliative care, with the added cultural difficulty that palliative care is often not viewed positively by Syrian patients. One example of how to avoid this tragic trade-off is the Jordan Breast Cancer Programme (JBCP), a national early-detection programme started in 2003 with the aim of improving the survival rate and decreasing the costs of treatment for breast cancer by lowering the stage at which Jordanian women are diagnosed with breast cancer from stages (III and IV) to stages (0, I and II) [11]. Enrolling female refugees within this programme could have a high impact on decreasing breast cancer mortalities [38].

### Strengths and limitations of this study

An important strength of this study lies in the deep knowledge of participants about the organizational structure of cancer care delivery among the refugee community, the recruited humanitarian actors, and rich understanding of Jordanian and Syrian refugee culture. The principal investigator is Syrian and has working experiences in the humanitarian sector, lending depth of understanding to the context, non-verbal cues, and authentic understanding of organisational structure and cultural issues in the context of Syrian refugees in Jordan [39,40]. This insider perspective was balanced with the involvement of outside researchers in the analysis to maintain the objectivity in interpretation of findings. However, one of the limitations of the study is the sample size. While using a small sample size is typical of expert stakeholder studies on focused topics, we cannot infer that the views expressed here reflect the wider refugee health provider and policy community. Despite this limitation, the specification of the research questions and the limited number of health actors working with Syrian cancer patients in Jordan led to the attainment of theoretical saturation in most of the questions raised and all of those reported here. A key limitation of this study is the absence of patients’ direct perspectives who were not recruited due to the challenges of ethical approval and the time sensitive nature of the research question.

### More research needed

This research aims to pave the way for further studies in refugee cancer care management and policy development to improve equitable access to cancer care. Patient and family perspectives will be critical to developing sensitive strategies and policies. Reflecting the experiences of cancer patients in future research would help to better understand their material, physical and emotional constraints in accessing available services, and it would increase knowledge about the consequences of current cancer policy on the emotional well-being of patients and its impact on the refugee household. This would be valuable data for informing and revising current policy based on field realities and refugee’ needs. It will also be important to further explore the cultural barriers to palliative care from the perspective of both health care providers and patients. This was proposed by our expert cohort and given its significance, is worth investigating further. The results of such research would help to maximise the intended outcomes of any future interventions and programmes to provide palliative care for refugee patients.

Finally, both the literature review and expert participants noted lack of data on the prevalence of cancer among refugee populations as a main challenge for cancer policy development. This confirms the need to obtain further data about the prevalence of cancer among refugee populations and the survival rate using quantitative research methods will help to formulate effective cancer care policies and improve the cost-effectiveness of interventions. This could have a positive impact both in encouraging donors to invest in cancer care, and in helping policy makers to formulate sustainable strategies to address cancer among refugees [41].

### What can be done now? Improvements within existing systems

There is a great need to expand the current funding and decrease bureaucratic barriers for referral and treatment for refugees with cancer, for screening, treatment, and palliative care. More sustainable and predictable funding commitments would enable health actors to provide a complete and continuous cancer treatment for refugees, and it would help create a systematic referral pathway for cancer patients that prevent some reported dramatic situations where patient treatment was set on hold while waiting for funding.

While increased funding will require political negotiations and international commitment, there are several immediate, low cost improvements that can significantly improve patient care. Although there is a system for coordination and information sharing between health actors in Jordan, our data revealed that there are nonetheless important challenges that urgently need to be addressed to improve coordination and knowledge sharing. Foremost would be to improve key stakeholders’ awareness of different health actors, projects and programmes in the region and available resources and existing referral pathways across their respective programmes. A coordinated and regularly updated mapping of services and rapid dissemination to key agencies and hospital officials and staff would help to shorten the referral path for patients and facilitate access to available services for early stage cancers. This would increase patients’ chances to obtain subsidized care, and increase cancer survival. For advanced cancers, better coordination between screening, diagnosis and referral to available palliative care services could improve access to more appropriate clinical support for symptom relief as well as compassionate, dignified care for terminal cancer patients and their families.

## Conclusions

In this paper, we investigated cancer care for Syrian refugees in Jordan to shed light on both a neglected population in research and a neglected disease in the humanitarian response. We have confirmed existing and identified new challenges for policy makers in planning and providing cancer care for refugees in this middle-income setting. These include the lack of data on cancer prevalence among refugees and decreased funding commitments for hosting countries that continue to have a significant impact on refugees’ lives and the ability of clinicians to meet basic obligations of care, and resulting high levels of moral distress for providers and policy makers.

Unintended harms seem to emerge from several aspects of the Syrian-Jordanian refugee sitation, including i) the implementation of short term funding schemes—designed to address infectious disease that generally require shorter and cheaper treatments than cancer—in long term refugee settings; and ii) the imposition of regulations restricting refugees’ movements includung access to health care by hosting countries, through the use of measures such as the MOI card and asylum certificates. Refugees’ family links in this setting seemed to play an important role in covering their unmet health needs, highlighting the potential for existing socioeconomic inequities to be widened within current cancer care policies. Findings revealed an ethical tension between health care providers duty of care to cancer patients and policy makers’ responsibility to make decisions to maximise public health benefit, and highlight coping strategies used by health care providers to support cancer patients such as establishing informal donation platforms and indirect referral pathways. The severity of the moral distress described suggests a need for better support for frontline clinicians.

More research around policy and service delivery for chronic health conditions such as cancer is urgent and critical to building effective public health strategies for long-term refugee populations in intractable political situations. Within this agenda, we argue that efforts to strengthen existing cancer care policy for refugees are essential to the well-being and dignity of those who cannot pay for treatment, and for those who take responsibility for decision-making and providing care at the national policy level and on the frontlines of clinical and humanitarian care.

## Supporting information

S1 Table Study participants. The letter in each interviewee code refers to an institution, and the number to an individual interviewee from the institution in question. There were four different institutions in total (which we denote as A, B, C and D).
(PDF)

S1 File Study protocol cancer care. (PDF)

S2 File Record of written consent. (PDF)

S3 File Question guide, english & arabic, cancer care. (PDF)

S4 File Participant information sheet. (PDF)
