# Supplementary material for: "If I have a cancer, it is not my fault I am a refugee”: A qualitative study with expert stakeholders on cancer care management for Syrian refugees in Jordan
Source: PLoS One. 2019 Sep 27;14(9):e0222496. doi: 10.1371/journal.pone.0222496 (PMC6764666; doi:10.1371/journal.pone.0222496)
Supplement: S1 Table — The letter in each interviewee code refers to an institution, and the number to an individual interviewee from the institution in question. There were four different institutions in total (which we denote as A, B, C and D). (PDF) [file pone.0222496.s001.pdf]

S1 Table. Study participants. S1 Study participants.

Interviewee Code	Position	Gender
A1	Health Care Provider	Male
A2	Policy Maker	Male
A3	Health Care Provider	Male
A4	Health Care Provider	Female
A5	Policy Maker& Health Care Provider	Female
A6	Health Care Provider	Male
A7	Policy Maker& Health Care Provider	Male
B1	Policy Maker	Male
B2	Policy Maker	Female
B3	Policy Maker	Male
C1	Health Care Provider	Male
D1	Policy Maker	Male
