# Supplementary material for: "If I have a cancer, it is not my fault I am a refugee”: A qualitative study with expert stakeholders on cancer care management for Syrian refugees in Jordan
Source: PLoS One. 2019 Sep 27;14(9):e0222496. doi: 10.1371/journal.pone.0222496 (PMC6764666; doi:10.1371/journal.pone.0222496)
Supplement: S1 File — (PDF) [file pone.0222496.s002.pdf]

EVALUATION OF CANCER MANAGEMENT FOR SYRIAN REFUGEES IN JORDAN

02 May 2016

RESEARCHER:
Manar Marzouk
MSc in International Health and Tropical Medicine,
Nuffield Department of Medicine, University of Oxford

PLACEMENT SUPERVISORS
Dr Slim Slama, MD, MPH, Medical Officer.
slamas@who.int

Dr Ibtihal Fadhil, Regional Adviser. fadhili@who.int

ACADEMIC SUPERVISOR:
Dr Gail Carson, Head of the ISARIC Coordinating Centre. gail.carson@ndm.ox.ac.uk

Contents

ABBREVIATIONS 4
1. Study Title: 5
2. Principal investigator: 5
3. Placement supervisors: 5
4. Academic supervisor: 5
5. Placement location: 5
6. SYNOPSIS 6
7. BACKGROUND AND RATIONALE: 7
Overview: 7
Why cancer research among Syrian refugees? 7
Cancer Registry and Speculation of hidden burden of cancer among Syrian refugees: 8
Development of cancer care for refugees in Jordan: 9
Cancer Care 9
Ethical Issues: 10
8. OBJECTIVES AND ENDPOINTS 10
9. STUDY DESIGN 11
What are the barriers to access cancer care among Syrian refugees in Jordan? 11
Does lack of access to care create a conflict between duty of care and distributional justice among cancer care providers? 11
10. PARTICIPANT IDENTIFICATION 11
Study Participants 11
Inclusion/ exclusion criteria 12
11. STUDY PROCEDURES 12
Recruitment for the interviews: 12
Definition of End of Study 12
12. SAFETY REPORTING 13
13. DATA ANALYSIS AND STATISTICS 13
14. DATA MANAGEMENT 13
Access to Data 13
Data Recording and Record Keeping 13
15. QUALITY ASSURANCE PROCEDURES 13
16. ETHICAL AND REGULATORY CONSIDERATIONS 13
Declaration of Helsinki 13
Approvals 13

Ethical Issues Mitigation: ... 13
17. FINANCE AND INSURANCE ... 14
Funding ... 14
Insurance ... 14
18. PUBLICATION POLICY ... 15
19. BIBLIOGRAPHY ... 15

ABBREVIATIONS

WHO	World Health Organisation
MSF	Doctors without Borders
KHCC	King Hussein Cancer Centre
KHCF	King Hussein Cancer Foundation
ECC	Exceptional Care Committee
UNHCR	United Nations High Commissionaire for Refugees
EMRO	World Health Organisation Regional Office for the Eastern Mediterranean
MoH	Ministry of Health

1. Study Title:

Analysing Barriers in Accessing Cancer Care among Syrian Refugees in Jordan

2. Principal investigator:

Manar Marzouk
MSc International Health and Tropical Medicine
Nuffield Department of Medicine
University of Oxford
Old Road Campus
Oxford OX3 7LF

3. Placement supervisors:

Dr Ibtihal Fadhil
Regional Adviser, Noncommunicable diseases
World Health Organization
Regional Office for the Eastern Mediterranean
P. O. Box 7608
Nasr City (11371)
Cairo, Egypt

Dr Slim Slama
MD, MPH, Medical Officer, Noncommunicable diseases
slamas@who.int
World Health Organization
Regional Office for the Eastern Mediterranean
P. O. Box 7608
Nasr City (11371)
Cairo, Egypt

4. Academic supervisor:

Dr Gail Carson
Head of the ISARIC Coordinating Centre
gail.carson@ndm.ox.ac.ukCentre for Tropical Medicine and Global Health, University of Oxford
Nuffield Department of Medicine Research Building
Old Road Campus
Oxford OX3 7LF

5. Placement location:

World Health Organization
Regional Office for the Eastern Mediterranean
P. O. Box 7608
Nasr City (11371)
Cairo, Egypt

Centre for Tropical Medicine and Global Health, University of Oxford
Nuffield Department of Medicine Research Building
Old Road Campus
Oxford OX3 7LF

6. SYNOPSIS

**Study Title**	Analysing Barriers in Accessing Cancer Care among Syrian Refugees in Jordan	
**Study Design**	Qualitative	
**Planned Sample Size**	- 4-6 Members of ECC committee within UNHCR, Jordan<br>- 4-6 Health workers / Officers within MSF, Jordan<br>- 4-6 Oncologists within King Hussein Cancer Centre<br>- 2 Heath Officers / Policy makers with WHO Jordan and/or EMRO office in Egypt	
**Planned Study Period**	2 May – 15 July 2016	
**Primary**	**Objectives**	**Endpoints**
	To explore the main barriers preventing Syrian refugees from accessing cancer care in Jordan (through literature review and qualitative research). Focusing on screening and detection, treatment, and palliative care	Thematic analysis of main obstacles that impact access to screening among Syrian refugees in Jordan
		Thematic analysis of main obstacles that impact access to treatment among Syrian refugees in Jordan
		Thematic analysis of main obstacles that impact access to palliative care among Syrian refugees in Jordan
**Secondary**	To explore the ethical conflict among cancer care providers between duty of care and distributional justice in the Syria refugee setting in Jordan(using qualitative research methods)	Thematic summary of the conflict between distributional justice and duty of care among cancer care providers
	To understand challenges in improving cancer care among Syrian refugees in Jordan	Thematic summary of health worker's suggestions to improve current cancer care for refugees in Jordan

7. BACKGROUND AND RATIONALE:

Overview:

A notable increase in the burden of non-communicable diseases, such as cancer, was reported between 1990 and 2013 by the global burden of disease registry. [1] In 2013, cancer was considered by WHO to be the second leading cause of death among non-communicable diseases, accounting for 8.2 million mortalities and more than 14 million new cases worldwide in 2012.[2] More than two thirds of cancer deaths occur in low and middle-income countries.[2] The low survival rate in such settings is strongly associated with poor access to treatment, late detection, and limited access to screening.[3] Moreover, the curative probability is high worldwide among many common cancers, such as breast and colorectal cancer. Leukemia among children also has a high curative rate if adequate treatment is provided at an early stage. [3] The delay in addressing these diseases is associated with a decrease in the quality of life and productivity, an increase in the expenditure on treatments if preventable cancers are undetected by screening, and an increase in the mortality rate caused by cancer.[4] Palliative care in cancer management is essential in improving the quality of life. It helps to provide a relief from physical and psychological problems among 90% of advanced cancer patients. Community and home-based care could play an important role in provision of relief and palliative care for patients and their families in low income settings.[3]

Refugee situations could add further delay to the provision of cancer care for patients. Especially in low and middle- income countries, where refugee cancer care is neglected. [5] The recent Syrian refugee crisis raised many concerns about cancer management in the hosting countries such as Lebanon and Jordan. A survey conducted by UNHCR indicated that seeking medical care is the reason behind the return of 11% of refugees to Syria despite the risk to their life. [6]

Stretched health resources and poor funding are among the main challenges facing hosting countries in addressing cancer among Syrian refugees. Prior to the Syrian crisis these countries had struggled to meet the increased demand on cancer care for their own citizens. For instance, the burden of cancer among Jordanian citizens had stressed the Jordanian health system in terms of funding and health workforce. Improving resource allocation could increase the survival rate. Some cancer treatments may be extremely expensive, however, low-cost and effective treatment options are currently available for many malignancies, such as breast cancer and childhood leukemia, two of the most common cancers among Syrian refugees.[7] Although the consequentialist approach could be justified where there are limited resources, it may raise many ethical concerns such as fairness and conflict between distributional justice and duty of care.

In this research, I will conduct qualitative research using a semi-structured interview method to analyse the main barriers preventing refugees from accessing cancer care, using the Syrian refugee population in Jordan as a case study. Due to the limitations of research time, I will focus my research mainly on screening, treatment and palliative care. In addition, I will restrict my research to four type of cancers, which are considered to be the most common; breast cancer, colorectal cancer, lung cancer and childhood leukemia. I will also restrict my interviews to the three main cancer care providers and decision makers in the Syrian refugee context in Jordan, which are: UNHCR, including members of the exceptional care committee who select eligible cancer patients for treatment, King Hussein Hospital, the main cancer treatment provider for eligible refugees, and MSF, one of the main primary care providers in an emergency context. Finally, during the interviews I will embed ethical analysis regarding the conflict between duty of care and distributional justice and fairness analysis.

Why cancer research among Syrian refugees?

The change in displacement trends requires a development of the humanitarian response in order to meet the new emerging health needs among refugees. The current humanitarian response strategy is based on experiences from refugee camps in Sub-Saharan Africa, where communicable diseases account for the main causes of death, while the recent refugee crisis in the Middle East, induced by conflicts in Iraq and Syria, has raised different health concerns, as the prevalence of non-

communicable diseases tends to be higher in the middle-income countries such as Syria and Iraq compared with low-income countries. [5] Cancer was the second leading cause of deaths among the Syrian population before the war according to WHO global health observatory. Even during the war, cancer cases witnessed a notable increase. In 2012 it was still considered one of the main causes of death in Syria. [8] There is limited data about the burden of diseases in Syria in the past 3 years.
Worldwide, the estimated length of stay in the country of refuge has increased from 9 years in 1993 to 13 years in 2003. The Syrian crisis is becoming a long term crisis after 5 years of conflict and displacement.[9] The refugee situation is becoming a durable one and we may anticipate that the burden of non-communicable diseases is more likely to be higher than communicable ones following the global epi-transitional trend of the burden of diseases. For instance, Iraqi refugees have been refugees in the Middle East since 2003. [5]

Cancer Registry and Speculation of hidden burden of cancer among Syrian refugees:

Limited updated data about the burden of cancer among Syrian refugees:

One of the main limitations of research on cancer among Syrian refugees is the scarcity of available data on incidence, survival, and mortality. Monitoring of the incidence and prevalence of cancer among this population are hindered by the lack of a universal registry system for the refugee population, limited access to health care, and the displacement situation itself. [2] The below Figures provided by WHO about cancer incidence in Syria are based on national incidence estimates from neighboring countries.[10] There is no mortality data available. Figures are based on national incidence estimates and modelled survival. [10]
However, due to the limitations of research time, I have used the available data as a basis to restrict my research study to the most common cancers among this population. This would include breast cancer among women, colorectal cancer among both women and men, lung cancer among men and Leukemia among children.

Figure 1: Cancer Incidence in Syria (2014)

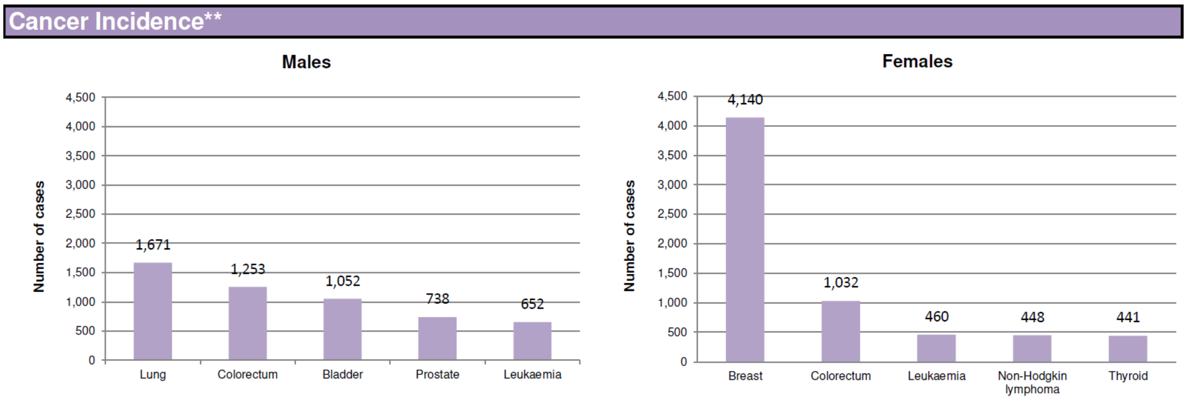

Source: (WHO,2014)[10]

Table 1: Priority cancer type for the study

Adult		Children
Male	Female	
Lung/ Colorectal	Breast/ Colorectal	Leukaemia

Registration Process:

The current available data impose two main challenges. First, there is no recent data about the burden of cancer among this population in the past 3 years. Second, the registration process of Syrian refugee cancer patients may hide potential patients such as those who are not registered within UNHCR, as refugees, or patients with cancer who potentially had not yet been diagnosed due to poor access to screening and detection services. In this research, I will use all available and accessible data from WHO, UNHCR, MSF, and King Hussein hospital, and if possible the Jordanian Ministry of Health, in order to establish the current burden of cancer among Syrian refugees, and show the cancer profile among this population.

Moreover, through qualitative research using an individual interview method with the ECC committee and King Hussein hospital, I will highlight the process of cancer patient registration, challenges and potential gaps in the registration.

Development of cancer care for refugees in Jordan:

In 2011, 134 Syrian refugees presented with cancer to the Jordanian health clinic, the majority of which were children with Leukemia. The number increased to 188 cases in 2012, and it is estimated that the number will continue to increase following the constant influx of Syrian refugees.[11]

Establishment of Exceptional Care Committee in Jordan:

In order to address the gap in cancer care provision, UNHCR has established an Exceptional Care Committee in Jordan, serving as a triage system. The main role of this committee is to identify refugees with cancer who fulfil the criteria of eligibility for treatment. Many Syrian refugees have benefited from health care facilitated by this committee. Yet, there are many concerns about patients who were not eligible either because of a poor prognosis or limited funding.[5,12] The committee plays a role in addressing the increase of identified cancer patients among Syrian refugees. In the period 2011-2012, 511 applications for cancer treatment were reviewed by the ECC. [5]More than 50% of applications were denied treatment due to poor prognosis or high cost of treatment. The mean amount requested by each cancer application was $11,540, while the mean amount of accepted application was $4,626.[9]This raises many ethical issues regarding the selection criteria, how the decisions are being communicated, and patients' rights to challenge the process.[10]

There is limited research about patients with cancer who are not covered by this committee. Researching and analyzing current health policies of organizations working with refugees and hosting countries may provide solutions to fill gaps in cancer management in Lebanon and Jordan.

In this research, I will illustrate the main challenges and gaps in provision of cancer care for Syrian refugees in Jordan.

Cancer Care

1. **Treatment Provision:**

Currently the eligible refugees for cancer care by ECC are provided with treatment through King Hussein Hospital in Jordan, which has a specialist cancer center in Amman, Jordan.[13] The cost is covered through donations made to its goodwill fund. However, there are limited data available about the treatment prioritization, the cancer prioritization, and the alternative care provided in case of partial funding.[12] In this research, I will try to provide a comprehensive view of the treatment provision

for Syrian cancer patients through King Hussein hospital, using qualitative methods by semi-structured interviews with health–decision makers at the center, and principle oncologists with direct contact with the referred patients from ECC (UNHCR,2013).[12]

2. **Palliative and post-discharge care:**

There is scarce information about the palliative and post discharge care provided for Syrian refugees during and following the treatment on a community and primary care level. As MSF is one of the main primary care providers in the refugee context in Jordan, I will conduct individual interviews with health care providers through MSF, and the main decision maker in order to enrich scarce data about the palliative and post discharge care for Syrian cancer patients (MSF, 2014).[14]

Ethical Issues:

Underfunding and resource constraints impose pressure on decision makers regarding resource allocation to achieve the greatest good for the greatest number of people.[9]

Although the consequentialist approach, which suggests that individual interests are sacrificed for the collective benefit, may be justifiable in a refugee context, this approach raises two ethical issues. First, the tension between the duty of care and distributional justice raised among health care providers, which was used in other contexts to inform policy change, such as provision of HIV treatment in Africa by MSF "It is not only a matter of a doctor in a remote area situating themselves within the broader landscape of international humanitarian needs—the stream of influence can run from the bottom up. It was the voice of frustration of doctors not being able to treat HIV that convinced MSF to launch treatment programmes across Africa."[15]

A second ethical problem that could emerge is fairness, and respect of patients. The method used to communicate to people affected by the decision would be crucial in keeping public trust even in a situation where policies breach some moral consideration.[12] It also guarantees that the trade-offs are being decided openly and take into consideration individuals' fundamental well-being and values. [12]

Another issue is patient's eligibility for alternative care, and their right to challenge the decision, the process for reviewing challenges, and how the policy is being communicated to patients and their families. All these issues require consideration from policy makers in order to assure openness and high accountability toward patients.[13]

In this research, I will provide ethical analysis of the conflict between distributional justice and duty of care through individual interviews with health care providers working with UNHCR, MSF, and KHC who are in direct contact with Syrian refugee cancer patients. While through interviews with ECC committee members, I will evaluate the fairness in decision making by the committee, the commutation method used, and the availability of any alternative care.

8. OBJECTIVES AND ENDPOINTS

**Primary**	**Objectives**	**Endpoints**
	To explore the main barriers preventing Syrian refugees from accessing cancer care in Jordan (through literature review and qualitative research). Focusing on screening and detection, treatment, and palliative care	Thematic analysis of main obstacles that impact access to screening among Syrian refugees in Jordan
		Thematic analysis of main obstacles that impact access to treatment among Syrian refugees in Jordan
		Thematic analysis of main obstacles that impact access to

		palliative care among Syrian refugees in Jordan
**Secondary**	To explore the ethical conflict among cancer care providers between duty of care and distributional justice in the Syria refugee setting in Jordan (using qualitative research methods)	Thematic summary of the conflict between distributional justice and duty of care among cancer care providers
	To understand challenges in improving cancer care among Syrian refugees in Jordan	Thematic summary of health worker's suggestions to improve current cancer care for refugees in Jordan

9. STUDY DESIGN

We propose a qualitative study design. We will conduct semi-structured individual interviews as follows:

- 4-6 Members of ECC committee within UNHCR, Jordan
- 4-6 Health workers/Officers within MSF, Jordan
- 4-6 Oncologists within King Hussein Cancer Center
- 2 Heath Officers or Policy makers with WHO, Jordan or/and EMRO office in Egypt

Interviews will be led by the investigator as she is a native Arabic speaker, and in addition she speaks English. Questions will be translated into Arabic for interviewees who speak Arabic. The participant's response will be translated into English following the interviews. The interviews will also be audio recorded for future reference.

The questions listed below will be used as a prompt to guide the discussions to answer the research questions.

What are the barriers to access cancer care among Syrian refugees in Jordan?

- ✓ Access to screening and detection programmers
- ✓ Access to treatment at KHC
- ✓ Access to palliative care at KHC/ or through NGOs (MSF/IRC)

Method: semi-structured interviews with members of ECC within UNHCR, MSF health workers.

Does lack of access to care create a conflict between duty of care and distributional justice among cancer care providers?

- ✓ What is the potential conflict between duty of care and distributional justice among cancer care providers?

Method: Semi-structured interviews: UNHCR, KHC & MSF

10. PARTICIPANT IDENTIFICATION

Study Participants

The participants in this study will be members of the ECC with UNHCR in Jordan, health officers/ workers with MSF Jordan, Oncologists and policy makers at King Hussein Cancer Center in Jordan. They will be asked to sign a consent form and there may be a need for the head of the department/organisation to give approval. They will be contacted through a personalised email or

letter. They will be informed about the topic of the research and, if requested, a list of interview questions will be sent before the interview.
The EMRO office in Egypt will also be contacted in order to obtain data related to the burden of cancer among Syrian refugees in Jordan, such as mortality, survival, and incidence. The organisation will be contacted by email or via Skype. Interviewees are chosen because of their affiliation with an organisation/ institution working on provision/ managing cancer care for Syrian refugees in Jordan. Therefore, they might be guarded during interviews as their statements might be seen as reflecting the views of the institution and not their own. Anonymity should be offered as an option and be guaranteed if chosen. However, it might be difficult to maintain anonymity of interviewees because the institutions/ organisations where they are employed have a limited number of experts and therefore they might be recognisable by persons in the same field. This will be an important aspect to focus on during the writing of analysis of data and findings.
On the other hand, it is possible that participants are obliged by senior colleagues to take part in research. This questions their full consent to the interview. Therefore, the researcher will try to choose as participants only persons who can make an individual decision on whether or not to participate in the study. Although this might not always be possible, as it is difficult to understand the internal dynamics of the institutions. Participants will be given the option of reading the outcome of the analysis of the interviews if they so require and to withdraw from the study at any point in time if they wish.

Inclusion/ exclusion criteria

Inclusion criteria for health care staff (subgroup 6) are:

- No age restrictions
- Able and willing to participate

11. STUDY PROCEDURES

Recruitment for the interviews:

Informed Consent

Informed consent will be sought from all participants prior to engaging them in the interviews. Written and verbal versions of the Informed Consent Form will be shared by email with the participants detailing the nature and purpose of the study and what it will involve for the participant. It will be clearly stated that the participant is free to withdraw from the study at any time for any reason, and with no obligation to give the reason for withdrawal.

Discontinuation/Withdrawal of Participants from Study

Participants will have the right to withdraw from the study at any time. In addition, the investigator may discontinue a participant from the study at any time if considered necessary for any reason including ineligibility arising during the study or withdrawal of consent. If a participant withdraws part-way through the interview, she/he will be asked if the information already provided can be included in the analysis. If a participant wishes to withdraw, she/he will not be required to provide a reason for doing so. If she/he chooses to provide a reason for leaving the study, this will be recorded in the write-up of the project.

Definition of End of Study

The study will end as soon as the data collection and analysis is competed. The end point will be identified when data saturation is reached. This refers to the situation in data analysis where participants' descriptions become repetitive and confirm previously collected data. At that point data analysis is complete and data collection is terminated. Nevertheless, in the real world this

status of saturation may not be reached. However, the researcher will maintain transparency about the findings and challenges during the study.

12. SAFETY REPORTING

None identified.

13. DATA ANALYSIS AND STATISTICS

Data from interviews will be reviewed after each day of data collection. Systematic data analysis will be conducted after all qualitative data collection has been completed. Thematic analysis will be used to identify and code themes emerging from the data. The analysis and coding of the data will be discussed with the placement and academic supervisor.

14. DATA MANAGEMENT

Access to Data

Direct access will be granted to authorised representatives from the host institution for monitoring and/or audit of the study to ensure compliance with regulations.

Data Recording and Record Keeping

Paper notes and questions will be used during data collection. Each participant will be given a unique identifier number. Results will be entered into Microsoft Excel and saved as a password-protected file on the PI's work computer. As soon as data have been entered onto the computer, all paper copies will be destroyed using a paper shredder available for confidential waste on-site at the University of Oxford or EMRO office.

15. QUALITY ASSURANCE PROCEDURES

The study may be monitored or audited in accordance with the current approved protocol, ICH GCP, relevant regulations and standard operating procedures.

16. ETHICAL AND REGULATORY CONSIDERATIONS

Declaration of Helsinki

The Investigator will ensure that this study is conducted in accordance with the principles of the Declaration of Helsinki.

Approvals

The protocol, informed consent form, and the questions to be used will be submitted to OXTREC for written approval. The PI will submit and, where necessary, obtain approval from the above party for all substantial amendments to the original approved documents.

Ethical Issues Mitigation:

Interviewees are chosen because of their affiliation with cancer care for Syrian refugees in Jordan (UNHCR, KHC, and MSF). Therefore, they might be guarded during interviews as their statements might be seen as reflecting the views of the institution and not their own. Anonymity should be offered as an option and be guaranteed if chosen.

✓ Confidentiality:

Participant identity will be anonymized. However, in some cases there may be a need to convey some information that may link to the individual. For general information (insensitive) the approval of the individual will be obtained.

For sensitive information that may be provided by some staff, this will be taken into consideration and will be revealed anonymously and without any link to their identity.

(Measuring the sensitivity of the information will be discussed with my three academic and placement supervisors and with the participant.)

- ✓ Anonymity:

It might be difficult to maintain the anonymity of interviewees because the institutions where they are employed have a limited number of experts and therefore they might be recognizable by persons in the same field. This will be an important aspect to focus on during the writing of analysis of data and findings.

- ✓ Consent form

A valid consent form will be required from participants in the research.
However, it is possible that participants are obliged by senior colleagues to take part in research. This questions their full consent to the interview. Therefore, the researcher will try to choose as participants only persons who can make an individual decision on whether or not to participate in the study. Although this might not always be possible, as it is difficult to understand the internal dynamics of the institutions.

- ✓ Member Check:

A member check will be conducted during the interview process and at the conclusion of the study to increase the credibility and validity of the study, and at the same time to guarantee the ethical rights of participants. First, during the interview, the researcher will summarize information and then question participants to determine accuracy. Second, following the completion of the study, participants will be provided with the information that will be used, to be double checked and approved before it is reported to guarantee no damage to participants and their agreements. This will allow participants to critically analyse the findings and comments on them.
This technique is not perfect, but it is useful to reduce the incidence of incorrect data and the incorrect interpretation of data. The overall goal of this process is to provide findings that are authentic, original and reliable.

- ✓ Right to withdraw:

Participants will be given the option of reading the outcome of the analysis of the interviews if they so require and to withdraw from the study at any point in time if they wish.

- ✓ Reporting

Once the study is completed, the outcome of the research will be shared, by 15th August 2016, with EMRO office as a summary report of the data analysis, and with the Nuffield Department of Tropical Medicine in the form of a Masters Dissertation.

- ✓ Expenses and Benefits

No expenses or benefits will be provided for participants, as the interviews will take place by telephone. No transportation of participants will take place.

- ✓ Other Ethical Considerations

This study proposal will be submitted for ethical approval to the University of Oxford Tropical Research Ethics Committee (OxTREC).

17. FINANCE AND INSURANCE

Funding

All expenses of this study will be covered by the principle investigator's (CA) study budget from the MSc International Health and Tropical Medicine at the University of Oxford.

Insurance

The University has a specialist insurance policy in place which would operate in the event of any participant suffering harm as a result of their involvement in the research.

18. PUBLICATION POLICY

We will write up results of our research in a Masters course dissertation and submit a manuscript for publication. Authorship will be determined in accordance with the ICMJE guidelines and other contributors will be acknowledged.

19. BIBLIOGRAPHY

1. Murray, J.L.2014. Global, regional, and national incidence, prevalence, and years lived with disability for 301 acute and chronic diseases and injuries in 188 countries, 1990–2013: a systematic analysis for the Global Burden of Disease Study 2013. 386(9995):743-800. doi:10.1016/S0140-6736(15)60692-4.
2. WHO.2014. Global Status Report on non-communicable diseases 2014. World Health Organisation.176. doi:ISBN 9789241564854.
3. World Health Organization.2015. Cancer Fact Sheet. World Health Organisation. http://www.who.int/mediacentre/factsheets/fs297/en/. Accessed May 4, 2016.
4. Mojtahedzadeh, M. Figlin, R.A. Rosenfelt, F.P. Sherzai,D. Cooper, C.J & Nahleh, Z.A.2015. Literature Review and Profile of Cancer Diseases among Afghan Refugees in Iran : Referrals in Six Years of Displacement.3622-3628. doi:10.12659/MSM.895173.
5. Spiegel, P. Khalifa, A & Mateen, F.J.2014. Cancer in refugees in Jordan and Syria between 2009 and 2012: challenges and the way forward in humanitarian emergencies. Lancet Oncology. Doi: 10.1016/S1470-2045(14)70067-1.
6. Amnesty International UK. 2014. Syrian refugees in Lebanon forced to return to Syria because of lack of medical care - new report. https://www.amnesty.org.uk/press-releases/syrian-refugees-lebanon-forced-return-syria-because-lack-medical-care-new-report. Published 2014. Accessed February 14, 2016.
7. Farmer, P. Frenk, J & Knaul, F.M. et al. Expansion of cancer care and control in countries of low and middle income: A call to action. Lancet. 2010; 376(9747):1186-1193. Doi: 10.1016/S0140-6736(10)61152-X.
8. WHO. 2015. Syrian Arab Republic: WHO statistical profile. World Health Organisation. http://www.who.int/gho/countries/syr.pdf?ua=1.
9. The Guardian. 2016. What happened to history's refugees? http://www.theguardian.com/news/datablog/interactive/2013/jul/25/what-happened-history-refugees#Edict of Fontainebleau. Published 2013. Accessed February 16, 2016.
10. Assalman, I. Alkhalil, M & Curtice, M. 2011.Syrian Arab Republic: Cancer Country Profile. World Health Organisation. http://books.google.com/books?hl=en&lr=&id=M_y2iFtKRoUC&oi=fnd&pg=PA202&dq=Syrian+arab+republic&ots=C0BOpm0x4u&sig=xhlePJ76nMzutDL2p3R_n486w4M.
11. Murshidi,M.M. Hijjawi, M.Q.B.Jeriesat, S & Eltom, A. 2013. Syrian refugees and Jordan's health sector. The Lancet. doi:10.1016/S0140-6736(13)61506-8.
12. UNHCR. A Guide to UNHCR Supported Health Care Services in Jordan. UNHCR.
13. Smith, J. 2014. Cancer care for refugees. The Lancet Oncology. doi:10.1016/S1470-2045(14)70322-5.
14. Medecins Sans Frontieres. International Activity Report 2014: The Médecins Sans Frontières Charter. Med Sans Front. 2014:96.
