# Supplementary material for: "If I have a cancer, it is not my fault I am a refugee”: A qualitative study with expert stakeholders on cancer care management for Syrian refugees in Jordan
Source: PLoS One. 2019 Sep 27;14(9):e0222496. doi: 10.1371/journal.pone.0222496 (PMC6764666; doi:10.1371/journal.pone.0222496)
Supplement: S2 File — (PDF) [file pone.0222496.s003.pdf]

Manar Marzouk
manar.marzouk@ndm.ox.ac.uk
+44(0)7788287572
MSc International Health and Tropical Medicine
Nuffield Department of Clinical Medicine
NDMRB, University of Oxford, Old Road Campus,
Roosevelt Drive, Oxford, OX3 7FZ

Consent Form

STUDY TITLE: Management of Cancer Care for refugee populations: the case of Syrian Refugees in Jordan
RESEARCHER DETAILS: Manar Marzouk, PI

PURPOSE OF STUDY: The overarching aim for this qualitative study is to identify the main barriers preventing Syrian refugees from accessing cancer care in Jordan and examine the challenges for managing cancer care among refugee populations.
The results could inform policy on how to improve access by refugees to cancer care, such as screening, treatment, and palliative care. The results of this research could be used also in similar refugee settings to Jordan.

I hereby agree that

1. I have read the study information sheet, have had the opportunity to ask questions, and have received satisfactory answers

2. I understand that this project has been reviewed by, and received ethics clearance through, Oxford Tropical Research Ethics Committee (OxTREC).

3. I understand that my participation is voluntary and that I am are free to withdraw myself or my data at any time, without giving any reason, and without any adverse consequences

4. I understand who will have access to personal data provided

5. I understand how personal data will be stored (eg according to the Data Protection Act), and what will happen to the data at the end of the project

6. I understand how research will be written up and published

7. I understand how to raise concerns or make a complaint

8. I consent to being audio recorded

9. Understands that audio recordings may be used in research outputs (eg internal reports, journal publications)

Manar Marzouk
manar.marzouk@ndm.ox.ac.uk
+44(0)7788287572
MSc International Health and Tropical Medicine
Nuffield Department of Clinical Medicine
NDMRB, University of Oxford, Old Road Campus,
Roosevelt Drive, Oxford, OX3 7FZ

10. I agree to the researchers using direct quotes that will not identify me

11. I agree to the researchers using indirect quotes that will not identify me

12. I agree to take part in the study

participant initial

Name of Participant: ______________________

Signature: ______________________ Date: __________

Name of researcher: ______________________

Signature: ______________________ Date: __________
