# Supplementary material for: "If I have a cancer, it is not my fault I am a refugee”: A qualitative study with expert stakeholders on cancer care management for Syrian refugees in Jordan
Source: PLoS One. 2019 Sep 27;14(9):e0222496. doi: 10.1371/journal.pone.0222496 (PMC6764666; doi:10.1371/journal.pone.0222496)
Supplement: S3 File — (PDF) [file pone.0222496.s004.pdf]

Interview Guide: Cancer Care Management For Syrian Refugees in Jordan

Interviewer: Manar Marzouk
Arabic reviewer: Giorgia Ferrari (Arabic Lecturer at University of Exeter, UK)

Interview Guide

Introduction

<Introduction to research following “Procedure” section of Informed Consent Form>

Thank you for agreeing to participate in this study. I would like to learn from your professional experience and expertise about barriers for refugees to access cancer care, using Syrian refugees in Jordan as a case study.

Part I – About Yourself

Let us start with a few questions about yourself.

Guiding question: Who is my informant?

1. Name
2. Position / Job Title
3. Responsibilities
4. Length of working with the organisation/ Institution in a Syrian refugee context, and if any expertise from other refugee context

Part II –Overview of the cancer care among Syrian refugees in Jordan.

- ✓ Guiding question: Would you please tell me from your experience what cancer care provided for Syrian refugees through your organisation/institution?
- ✓ Can you tell me the history/rationale behind starting the provision of these services?

Follow up questions:

- ✓ Where these services are provided? (Through which partners)

Part III- Challenges in cancer care provision and management in a refugee setting, Syrian refugees in Jordan as a case study.

دليل المقابلة: إدارة رعاية مرضى السرطان للاجئين السوريين في الأردن

المقابلة: منار مرزوق
مدقق اللغة العربية: جورجيا فيراري (مدرسة اللغة العربية في جامعة أكستر، بريطانيا)

دليل المقابلة

المقدمة

تعريف بالبحث، ثم قسم الإجراءات المتضمن في استمارة الموافقة.

شكراً على موافقتك المشاركة في هذه الدراسة. أود أن أعرف من خلال خبرتك المهنية وتجاربك عن الصعوبات التي تواجه اللاجئين للوصول الى رعاية مرضى السرطان.

القسم الأول- تعريف عن نفسك

سنبدأ ببعض الأسئلة عن نفسك

1. أسئلة دليلة : من هو الشخص المقابل
2. الاسم
3. المهنة – المنصب
4. المسؤوليات
5. فترة العمل في المنظمة أو الجهة المقدمة للخدمات للاجئين السوريين، أو أي خبرة في مجال العمل مع اللاجئين.

القسم الثاني- لمحة عن رعاية مرضى السرطان للاجئين السوريين في الأردن.

- ✓ سؤال دليل: من خلال خبرتك، ماهي خدمات لرعاية مرضى السرطان المقدمة للاجئين السوريين من خلال جمعيتك أو مؤسستك؟
- ✓ هل تستطيع أن تخبرني عن تاريخ أو سبب البدء بتقديم هذه الخدمات؟

أسئلة إضافية

- ✓ أين تقدم هذه الخدمات؟ (من خلال أي من الشركاء)

القسم الثالث- الصعوبات المواجهة لدى تقديم خدمات رعاية واداره مرضى السرطان في أماكن اللجوء. دراسة حالة اللاجئين السوريين في لبنان.

Access to clinics/ hospital where cancer care is provided:<br>✓ Would you please describe the registration process of cancer patients and data collection method?<br>✓ How does it differ between patients? (In the community/ Camps/ Refugee status)<br>✓ Who has access to cancer care?<br>✓ What are the challenges you face in registration of cancer refugee patients?	*الوصول للمشافي والمستوصفات حيث يتم تقديم رعاية لمرضى السرطان*<br>✓ من فضلك، وضح عملية تسجيل مرضى السرطان والمنهج المتبع لجمع المعلومات<br>✓ كيف تميز بيت المرضى (في التجمعات، المخيمات، صفة اللجوء).<br>✓ من لديه وصول لرعاية السرطان<br>✓ ماهي الصعوبات التي تواجهك عند تسجيل اللاجئين المصابين بالسرطان.
Cancer Treatment<br>✓ Can you describe the process of providing cancer treatment for refugees?<br>✓ How cancer treatment is being prioritised?<br>✓ How the decision is being made?<br>✓ How the policy for cancer treatment was developed?<br>✓ What ethical argument would you raise to justify the current allocation for health resources among cancer patients?<br>✓ And what guidance/policy basis is being used to justify this method of allocation?<br>✓ What are the challenges in managing cancer treatment for refugee population?<br>✓ How are the patients and/or their families involved in the decision of their treatment?<br>✓ What do you feel about the current care provided?<br>i. What is working well in the provision of treatment for refugees? (Positive)<br>ii. What could be done to optimise the provision of this care?<br>iii. What prevent this from happening?<br>✓ In case of patients who are unentitled for treatment, how do you manage these cases?<br>✓ What are services provided for these patients? And what option do they have?<br>*Follow up questions* (UNHCR interviewee only)<br>✓ Would you tell me about the structure of ECC committee? (How members are being selected?)	*علاج السرطان*<br>✓ هل يمكن توضيح الية تقديم علاج السرطان للاجئين.<br>✓ كيف يتم تصنيف أولوية (تفضيل) علاج مرضى السرطان؟<br>✓ كيف يتم أخذ هذا القرار؟ (على أي أسس يتم اتخاذ هذا القرار؟)<br>✓ كيف تم تطوير سياسة علاج السرطان؟<br>✓ ماهي الحجج الأخلاقية التي تستخدمها لتبرير التوزيع الحالي للموارد الصحية لمرضى السرطان؟<br>✓ وماهي الأسس الدليلة أو السياسات المستخدمة لتبرير منهج التوزيع؟<br>✓ ماهي الصعوبات لإدارة علاج السرطان لفئة اللاجئين؟<br>✓ كيف يلعب المرضى أو عائلاتهم دورا في صنع القرار المتعلق بعلاجهم؟<br>✓ ماذا تشعر حيال الرعاية المقدمة حالياً؟<br>✓ ماهي الاشياء الإيجابية في العلاج المقدم للاجئين؟<br>✓ كيف يمكن تحسين الية تقديم هذه الخدمات؟<br>✓ ما لذي يمنع مثل هذا التحسين؟<br>✓ في حالة المرضى الغير مخولين بالعلاج، كيف يتم ادارة مثل هذه الحالات؟<br>✓ ماهي الخدمات المقدمة لهؤلاء المرضى؟ وماهي الخيارات المتوفرة لهم؟<br>أسئلة إضافية (لمقابلات العاملين في المفوضية)<br>✓ هل يمكنك اخباري عن بنية لجنة ecc؟ (كيف يتم اختيار أعضاء اللجنة)
Curative treatment (Out-patient care)	*العلاج في العيادات الخارجية*

✓ What happens to patients post discharge of the hospital/ clinic?	✓ ماذا يحصل للمرضى بعد تخريجهم من العيادة أو المشفى؟
✓ How these services is being prioritized?	✓ كيف يتم تفضيل تلك الخدمات؟
✓ How the treatment outcomes are measured and documented?	✓ كيف يتم تقييم نتائج العلاج وكيف يتم تدوينها؟
✓ Any data about surviving rate of those underwent the treatment?	✓ هل يوجد أي بيانات عن معدل الشفاء لدى المرضى الذين خضعوا للعلاج؟
✓ How the out-patients care policy is being developed?	✓ كيف تم تطوير السياسات المتعلقة برعاية المرضى خارج العيادات؟
✓ Can you say something about the ethical obligations that guide you in making decisions related to out-patient care?	✓ هل يمكنك أخباري عن الالتزامات الأخلاقية الضابطة لقراراتك المتعلقة بالرعاية خارج العيادات؟
✓ What are the challenges in out-patients care provision for refugee population?	✓ ماهي الصعوبات المواجهة عند تقديم الرعاية خارج العيادات لفئة اللاجئين؟
✓ What do you feel about the current care provided?	✓ ماذا تشعر حيال خدمات الرعاية المقدمة حالياً؟
What is working well in the provision of treatment for refugees?	✓ ما لأشياء الإيجابية حول علاج اللاجئين؟
iv. What could be done to optimise the provision of this care?	✓ كيف يمكن تحسين الية تقديم هذه الرعاية؟
v. What prevent this from happening?	✓ ما الذي يمنع مثل هذا التحسين؟
4- Palliative Care	*الرعاية التلطيفية*
✓ Can you describe the palliative care provided for Syrian refugees?	✓ هل يمكنك توضيح الرعاية التلطيفية المقدمة للاجئين السوريين؟
✓ How do you prioritise the provision of this care?	✓ كيف يتم تفضيل تقديم هذه الرعاية؟
✓ How the policy for palliative care was developed?	✓ كيف تم تطوير السياسات المتعلقة بالرعاية التلطيفية؟
✓ Can you tell me about the ethical obligations that have guided the development of these policy?	✓ هل يمكنك أخباري عن الالتزامات الأخلاقية الضابطة لقراراتك المتعلقة بالرعاية التلطيفية ؟
✓ What do you feel about the current care provided?	✓ ماذا تشعر حيال خدمات الرعاية المقدمة حالياً؟
✓ What are the challenges in providing this care? (And/or allocating resources for palliative care?)	✓ ماهي الصعوبات التي تواجهك عند تقديم هذه الرعاية؟ (و/أو عند توزيع الموارد للرعاية التلطيفية؟)
✓ What could be done to optimise the provision of this care?	✓ كيف يمكن تحسين الية تقديم هذه الرعاية؟
Part IV: Optimising Cancer Care	القسم الرابع: تحسين رعاية مرضى السرطان
✓ Are there policies making explicit mentioning of cancer care (from Registration to Treatment to Palliative care	✓ هل يوجد أي سياسات تذكر بشكل خاص رعاية مرضى السرطان (بدءا من التسجيل مرورا بالعلاج ثم الرعاية التلطيفية)
✓ What are the most pressing concerns in provision of cancer care for refugee population?	✓ ماهي المخاوف الأكثر الحاحاً المتعلقة بتقديم رعاية السرطان لدى فئة اللاجئين.
✓ From your involvement with managing the health response for refugees, Can you tell me	✓ من خلال خبرتك في إدارة الاستجابة الصحية للاجئين، هل يمكنك تقديم

through one example of challenge you face during your work (case study story)<br>✓ What can be done to improve the current cancer care for refugees?	أمثلة عن المصاعب التي تواجهها من خلال عملك (قصة عن دراسة حالة)<br>✓ ما الذي يمكن القيام به لتحسين الرعاية المقدمة حاليا للاجئين المصابين بالسرطان؟
Part V – Data and Contacts<br>*Lastly, I would like to know if you could recommend any contacts from the main health care providers in a refugee context who may be involved in cancer care.*<br><br>Guiding question: What other experts, documents, and data should I be aware of?<br>*follow up questions*<br>✓ Can you think of someone I should talk to with regard to my research?<br>✓ Can you recommend policy documents or data that give me a good overview about cancer care management among refugee population?	القسم الخامس- البيانات وجهات الاتصال<br>أخيرا، هل يمكنك نصيحتي بأي جهة اتصال يمكنك تقديمها لعاملين في قطاع الرعاية الطبية للاجئين والذين قد يكونوا ساهموا بتقديم رعاية السرطان.<br><br>سؤال دليل: هل يمكنك توجيهي لأي خبراء، ملفات، بيانات ينبغي أن أكون على علم بها؟<br>أسئلة إضافية<br>✓ هل بذهنك أي شخص ينبغي لي الحديث معه/معها بخصوص موضوع بحثي؟<br>✓ هل يمكنك أن تنصحني بملفات أو بيانات عن السياسات لإعطائي لمحة جيدة عن إدارة رعاية السرطان لدى اللاجئين ؟
Wrap-up<br>*We are coming to an end in our interview. Before we conclude, let me just ask you:*<br>Have I missed an aspect of cancer care management of cancer that you find particularly important?<br>Is there anything else you would like to share with me?<br><br>*Thank you so much for educating me about the cancer management among refugee population. Your responses have been very helpful for me.*	الختام<br>وصلنا الى نهاية مقابلتنا. قبل أن أختم، أوج أن أسئلك:<br><br>هل أغفلت أي ناحية متعلقة بإدارة رعاية السرطان والتي تعتقد باني يجب أن انتبه اليها؟<br>هل يوجد أي شيء اخر تود مشاركته معي؟<br><br>شكرا جزيلا لإغنائي بخبرتك عم إدارة السرطان لدى اللاجئين.<br>أجابتك كانت مساعدة جدا لي.

To whom it may concern

This is to confirm that the translation of the questions designed Manar Marzouk is an accurate representation of the questions in English.

Please do not hesitate to contact me should you have any queries.

Best Wishes,

Giorgia Ferrari

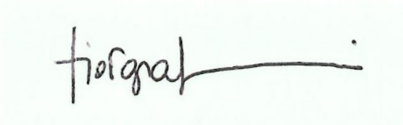

Lecturer in Arabic Language
Institute of Arab and Islamic Studies
University of Exeter

Telephone: +44 (0) 1392 72 4957
Email: g.ferrari2@exeter.ac.uk
