# Supplementary material for: "If I have a cancer, it is not my fault I am a refugee”: A qualitative study with expert stakeholders on cancer care management for Syrian refugees in Jordan
Source: PLoS One. 2019 Sep 27;14(9):e0222496. doi: 10.1371/journal.pone.0222496 (PMC6764666; doi:10.1371/journal.pone.0222496)
Supplement: S4 File — (PDF) [file pone.0222496.s005.pdf]

Manar Marzouk

manar.marzouk@ndm.ox.ac.uk
+44(0)7788287572

MSc International Health and Tropical Medicine
Nuffield Department of Clinical Medicine

NDMRB, University of Oxford, Old Road
Campus, Roosevelt Drive, Oxford, OX3 7FZ

Management of Cancer Care for Refugee Populations: the case of Syrian refugees in Jordan

PARTICIPANT INFORMATION SHEET

Dear [Title and Name],

I would now like to invite you to participate in this study: a series of semi-structured, qualitative interviews.

1. **Study title**
   Management of Cancer Care for refugee populations: the case of Syrian Refugees in Jordan

2. **Background and aims of the study**
   The overarching aim for this qualitative study is to identify the main barriers in accessing cancer care among Syrian refugees in Jordan, examine the challenges for managing cancer care among refugee populations, and investigate the ethical tensions between duty of care and distributive justice in the provision of cancer care among refugee populations.
   The results could inform policy on how to improve access by refugees to cancer care, such as screening, treatment, and palliative care. The results of this research could be used also in similar refugee settings to Jordan.

   The principal investigator for this study is Manar Marzouk, a candidate for masters in International Health and Tropical Medicine at the University of Oxford. (manar.marzouk@ndm.ox.ac.uk). You will be interviewed by the principal investigator, Manar Marzouk, and results will be analysed by the same person.

3. **Why have I been invited to take part?**
   I am conducting this qualitative study, consisting of a series of semi-structured interviews, which builds on the literature review on cancer management in emergency and refugee context but goes into greater detail with regards to the case of cancer care for Syrian refugees in Jordan in the period 2011-2016. We have noted that you or your institution was or is part of the health response

Manar Marzouk

manar.marzouk@ndm.ox.ac.uk
+44(0)7788287572

MSc International Health and Tropical Medicine
Nuffield Department of Clinical Medicine

NDMRB, University of Oxford, Old Road
Campus, Roosevelt Drive, Oxford, OX3 7FZ

strategy for Syrian refugees in Jordan. We would therefore like to ask you to consider participating in this qualitative research study.

4. **Do I have to take part?**
Your participation in this research study is voluntary and you may choose not to participate. Before agreeing to be interviewed, you could have the chance to read the semi-structured interview guide, and ask any questions you may have about the study. If you agree to participate, you may withdraw yourself and your data from the study without penalty at any time, and without giving a reason, by advising the researchers of this decision. Your identity and any information provided will be anonymised using an alphanumerical participant identifier and all data will be stored in a password protected electronic format on a secure server. We hope to record the interviews; however, you may choose not to be recorded – in which case we will be happy to document your interview with written notes.

5. **What will happen in the study?**
If you are happy to participate in the study, the researchers will confirm this by asking you to complete a written informed consent document before the interview. In this form, you may indicate whether you are happy to have the interview audio recorded, and whether you agree to the researchers using direct and indirect quotes that would not identify you in the final project report. Interviews will last between 30 minutes and 1 hour.

If possible, your interview will take place via Skype or by phone. Oral consent will be seek at the beginning of interview.

In the interview, you will be asked to provide your professional experiences, opinions, views and comments. Any personal views or comments made in the surveys will be kept anonymous, and you will have the opportunity to approve any results before publication and dissemination of outputs.

6. **Are there any potential risks in taking part?**
The interview may raise questions and issues that may be politically or otherwise sensitive, in that answers may be confidential or be perceived to affect the reputation, organisational or personal integrity, or emotional comfort of the study participants in any way. In order to mitigate any such risks, you will be offered anonymity for your participation.

As part of the consent process, you will be asked to consent to the use of direct quotes and indirect quotes which will not identify you. You should be aware of the possibility that the text of the final report could inadvertently reveal who you are, and of the possibility of being misquoted. However, the research team will mitigate these risks by giving you the opportunity to review and approve

Manar Marzouk

manar.marzouk@ndm.ox.ac.uk
+44(0)7788287572

MSc International Health and Tropical Medicine
Nuffield Department of Clinical Medicine

NDMRB, University of Oxford, Old Road
Campus, Roosevelt Drive, Oxford, OX3 7FZ

relevant parts and contexts within the final report before internal or external dissemination. There will be no direct benefit to you for participating in this research.

7. What happens to the research data provided?

Paper notes and questions will be used during data collection. Each participant will be given a unique identifier number. Results will be entered into Microsoft word and saved as a password-protected file on my work computer. As soon as data have been entered onto the computer, all paper copies will be destroyed using a paper shredder available for confidential waste on-site at the University of Oxford.

We expect to store the data as computer files for a period of five years. Data entered onto the computer will include the participant identifiers but no names or other identifying information. Only the core study person named above will have access to the research data collected for this study.

8. Will the research be published?

The results from this study will be disseminated in shape of a master's dissertation report for the University of Oxford. The data and analysis produced through this project may also be published in peer-reviewed journals, used to shape policy reforms, and inform the priorities and mechanisms of funders, in regards to cancer care in a refugee context.

9. Who has reviewed this project?

This project has been reviewed by, and received ethics clearance through, Oxford Tropical Research Ethics Committee (OxTREC).

The project is under the academic supervision of Dr. Gail Carson, clinical lead for ISARIC (gail.carson@ndm.ox.ac.uk).

This study is funded by Nuffield Department of Medicine, University of Oxford.

10. Who do I contact if I have a concern about the study or I wish to complain?

If you have a concern about any aspect of this project, please speak to the Principal Investigator, Manar Marzouk (+447788287572 manar.marzouk@ndm.ox.ac.uk) who will do her best to answer your query. The researcher should acknowledge your concern within 10 working days and give you an indication of how he/she intends to deal with it. If you remain unhappy or wish to make a formal complaint, please contact [Email: oxtrec@admin.ox.ac.uk , Address: Research Services, University of Oxford, Wellington Square, Oxford OX1 2JD]

Manar Marzouk

manar.marzouk@ndm.ox.ac.uk
+44(0)7788287572

MSc International Health and Tropical Medicine
Nuffield Department of Clinical Medicine

NDMRB, University of Oxford, Old Road
Campus, Roosevelt Drive, Oxford, OX3 7FZ
